# Rheumatoid shoulder assessed by ultrasonography: prevalence of abnormalities and associated factors

**DOI:** 10.11604/pamj.2016.24.235.9068

**Published:** 2016-07-13

**Authors:** Imane Elbinoune, Bouchra Amine, Moudjibou Wabi, Hanan Rkain, Souad Aktaou, Najia Hajjaj-Hassouni

**Affiliations:** 1 Rheumatology Departement, El Ayachi Hospital, Ibn Sina University hospital, Sale, Morocco; 2 Laboratory of Biostatistics, Clinical and Epidemiological Research (LBRCE), Faculty of Medicine and Pharmacy, Ibn Sina University Hospital, Rabat, Morocco; 3 LIPROS-URAC30, Mohammed V Souissi University, Rabat, Morocco

**Keywords:** Rheumatoid arthritis, shoulder, ultrasonography

## Abstract

**Introduction:**

The shoulder involvement in rheumatoid arthritis (RA) is common. It can be subclinical and compromise the function of the upper limb. Musculoskeletal ultrasonography can detect subclinical abnormalities in rheumatoid shoulder. Our aim was to assess the prevalence of ultrasound abnormalities in rheumatoid shoulder, and investigate their association with different parameters.

**Methods:**

Cross-sectional study including 37 patients with RA, meeting the ACR/EULAR 2010 classification criteria, who were enrolled during a month. A questionnaire with sociodemographic, clinical and laboratory data was filled in for all patients. Ultrasound evaluation was performed by a single experienced operator. For each patient, both of shoulders were evaluated.

**Results:**

Mean age was 50 years with female predominance. Median disease duration of RA was 7.5 years. All patients had a seropositive form of RA. Mean clinical DAS28 was 5.1. Mean HAQ was 1.2. Thirty-one (83.8%) patients had involvement of the shoulder: unilateral in 9(24.3%) cases and bilateral in 22(59.5%) cases. Synovitis was found in 16(43.2%) patients with Doppler in 4 (10.8%) cases. Sub-acromial bursitis was noted in 14 (37.8%) cases and the effusion in 20 (54.1%). Synovitis was noted especially in elderly individuals (p: 0.01). The Doppler was visualized in elderly patients (p: 0.01), with a shorter disease duration (p: 0.02) and with a high SDAI (p: 0.006). US inflammatory findings in anterior recess of glenohumeral joint were linked to a higher synovial index (p: 0.03) and a higher level of rheumatoid factor (p: 0.01).

**Conclusion:**

59.5% of our RA patients had bilateral involvement of the shoulder which was related to the disease activity. Ultrasound should be a systematic tool to look for the involvement of this joint in RA patients.

## Introduction

Shoulder involvement is frequent during the natural history of Rheumatoid Arthritis (RA). Several structures can be targeted by the disease; especially the glenohumeral joint but also periarticular structures. Only a small proportion of patients has clinically detectable shoulder tenderness and swelling, whereas up to 5% of patients after 2 years and 96% after 12 years show erosive damage at the shoulder [1, 2]. Thus, clinical evaluation of shoulder does not offer an accurate assessement of this joint [3]. Moreover, conventional radiography ascertains tardily the diagnosis. Owing to the necessity of early treatment in RA patients before the damage occurrence [4], musculoskeletal (MS) ultrasonography (US) has a great role in detecting subclinical abnormalities in rheumatoid shoulder in order to achieve a low disease status and eventually remission. It has proved to be a valid tool in the assessment of inflammatory arthritis, including RA, and to be more sensitive than clinical examination in such joints as rheumatoid shoulder. Also, it has shown a good correlation with clinical and biologic parameters [5, 6]. The aim of the present study was to evaluate the prevalence of abnormalities in rheumatoid shoulder, and to investigate their association with the clinical and laboratory measures.

## Methods

**Design and recruitement:** It was a cross-sectional study conducted in Rheumatology Department at El Ayachi Hospital (Sale-Morocco). Patients received clear informations about the purpose of this survey and agreed to participate with a verbal consent.

**Inclusion and exclusion criteria:** A total of 37 patients with RA, classified according to the 2010 ACR/EULAR classification criteria, consulting in our centre of rheumatology, were enrolled and recruited successively during the consultations done in June 2015. Patients with shoulder traumatism or surgery were excluded from this study.

**Baseline variables:** For each patient, a rheumatologist collected sociodemographic, clinical, and laboratory data: age, gender, BMI, disease duration, diagnosis delay, serological status of RA (according to Rheumatoid Factor RF and/or ACPA), pain intensity and global discomfort in a visual analogical scale, tenderness and swelling index, perfoming DAS28, CDAI and SDAI scores, HAQ, ESR, CRP and RF, ACPA levels. The presence of pain or limitation during different shoulder movements (flexion, extension, adduction, abduction and medial, lateral rotation) was noted. All patients gave written consent after receiving clear informations about the purpose of this cross sectional study.

**Ultrasonography assessement:** Shoulder evaluation with ultrasonography was achieved by a single experienced operator (senior rheumatologist) who was blinded to clinical findings, in the same day of clinical evaluation, using a Toshiba scanner, operating with a linear probe at 14 MHz. The scanning technique and the definition of pathology were established based on international guidelines OMERACT: synovial Hypertrophy is defined as an abnormal hypoechoic (relative to subdermal fat, but sometimes may be isoechoic or hyperechoic) intraarticular tissue that is non displaceable and poorly compressible and which may exhibit Doppler signal, and synovial Fluid or effusion as an abnormal hypoechoic or anechoic (relative to subdermal fat, but sometimes may be isoechoic or hyperechoic) intraarticular material that is displaceable and compressible, but does not exhibit Doppler signal. Bone Erosion is defined as an intraarticular discontinuity of the bone surface that is visible in 2 perpendicular planes [7]. Articular and periarticular shoulder structures were examined looking for synovial hypertrophy (SH) and effusion (SE) in anterior, posterior and inferior recesses of glenohumeral joint (GHJ) in B mode and Doppler, bursitis in subacromial/subdeltoid (SAD) regions, and erosions in humeral head and in greater and lesser tuberosities. Assessments of different recesses were done using standardized sections through both shoulder joints as following: ventral transverse section and ventral longitudinal section over the intertubercular sulcus, for visualization of the long biceps tendon and detection of fluid accumulations and detection of tenosynovitis; Ventral transverse section in the coracoacromial window in neutral position; ventral transverse section during maximal external and internal rotations; dorsal transverse section through the infraspinous fossa laterally below the scapular spine; axillary longitudinal section, for detection of synovitis, synovial proliferation, and erosion of the humeral head. Joint effusion, synovitis, and power Doppler signal in the synovial membrane of the shoulder were evaluated and classified on 4 grade semi-quantitative scales from 0 to 3 [8]:

**Scoring of B mode synovitis and effusion:** Grade 0 or normal: Normal joint: no synovial thickness, no joint effusion; Grade 1 or mild: Mild synovial hypertrophy without bulging over the line linking the tops of the bones / Minimal joint effusion; Grade 2 or moderate: moderate synovial hypertrophy over the line linking the tops of the bones but not extending to the diaphysis / Minimal joint effusion; Grade 3 or severe: severe synovial hypertrophy bulging over the line with extension to at least one diaphysis / Significant joint effusion.

**Scoring of power Doppler (PD) synovitis:** Grade 0: No PD signal; Grade 1: 3 isolated spots or 2 confluents spots or 1 confluent spot 2 isolated spots of signal; Grade 2: vessel signals in < 50% of the areas of the areas of the synovium; Grade 3: vessel signals in ? 50% of the areas of the synovium.

**Also, erosions were assessed semi-quantitatively from 0 to 3** [9]: Grade 0: normal cortical surface; Grade 1: abnormal bone cortical surface without visible defect in 2 shots; Grade 2 defect visible bone surface in two perpendicular planes; Grade 3: bone defect creating extensive bone destruction. Osteophytes were researched in inferior recess of GHJ. Acromio-clavicular joint (ACJ) was not evaluated since abnormalities at this region are frequently seen even in healthy subjects and might not reflect RA involvement.

### Statistical analysis

All statistical calculations were done by computer using SPSS software (SPSS, Inc., Chicago, IL, USA). Quantitative variables were expressed as means ± SD or as medians and interquartile range, depending on their distribution. For categorical variables, the percentages of patients in each category were calculated. Categorical variables were analysed using chi-squared tests, quantitative variables were analysed using the unpaired t-test for normally distributed ones, and Mann-Whitney U-test for non-normally distributed ones. The significance set was fixed at a p value of equal to or less than 0.05.

## Results

37 patients were included in this study and 74 shoulders were evaluated clinically and by US. 22 shoulders were painful. Patients had a mean age (SD) of 50.3 years (10.9) with female predominance (86.5%). Median disease (RA) duration was 7.5 months. All patients had a seropositive form of RA. 74% of patients were on DMARDs and 22% of them were treated with biologics (TNFa inhibitor, Rituximab). Corticosteroids were given to 93% of patients and 19% of them were taking NSDAIs. Table 1 reveals the demographic, clinical and biological characteristics of our population. At the level of the GHJ, Synovial hypertrophy was found in 16 (43.2%) patients, synovial effusion in 20 (54.1%), Doppler signal in 4 (10.8%). According to the number of shoulders, SH was noted in 27 (20%) shoulders, SE in 23 (17%), Doppler signal in 6 (4%). On the other hand, 29.7%, 29.7% and 54.1% of patients presented respectively with inflammatory involvement of anterior, inferior and posterior recesses of GHJ. SAD bursitis was noted in 37.8% of patients with a power Doppler in 16.2% of them. Erosion was found in 24 (64.9%) patients, with a grade 3 noted in 36.7% of them. Concerning osteophytes, they were visualized in 5 (13.5%) cases. In total, US inflammatory abnormality was found in 83.8% of patients; it was bilateral in 59.5% of them. Table 2 summarises the main inflammatory findings found in ultrasonography in our population. When we evaluated the association between clinical, biological characteristics and inflammatory findings in ultrasonography, patients with US inflammatory involvement of shoulder had a shorter median disease duration (p: 0.01). Presence of synovitis was noted especially in elderly individuals (p: 0.01). The power Doppler was visualized in elderly patients (p: 0.01), in patients with a shorter disease duration (p: 0.02) and when the disease had a high activity assessed by SDAI (p: 0.006). The SAD bursitis was not associated nor with a high SDAI (p: 0.02), nor with elevated ESR (p: 0.05) or CRP (p: 0.03) and nor with limited shoulder (p: 0.05). US inflammatory findings in anterior recess of GHJ were linked to a higher synovial index (p: 0.03) and a higher level of rheumatoid factor (p: 0.01). Table 3 shows associations between US findings and clinical, biological features.

**Table 1:** Baseline characteristics of patients

Patients (n)	37
Age, mean (SD), years	50.3±10.9
Female n (%)	32 (86.5)
Disease duration, median (IQR), months	7.5 [3.3, 19.3]
DAS28, mean (SD)	5.1±1.5
SDAI, median (IQR)	30.5 [19.6, 58.1]
CDAI, mean (SD)	20.6±13.2
HAQ, mean (SD)	1.2±0.7
EVA douleur, mean (SD)	51.4±20.9
RF, median (IQR), U/l	68 [28, 163.5]
ACPA, median (IQR), U/l	200 [200, 342.5]
ESR mm/h, median (IQR)	39 [22, 67.5]
CRP mg/l, median (IQR)	10 [4.3, 29.6]
Shoulder pain, n (%)	22 (59.5)
DMARDs, n (%)	20 (74)
Corticosteroïds, n (%)	25 (93)
Biologics, n (%)	6 (22)
NSAIDs, n (%)	5 (19)

**Table 2:** Prevalence of US inflammatory findings in the shoulders: data referring to the number of patients

	1 Shoulder	2 Shoulders	Total
**Glenohumeral joint**			
**Anterior recess**			
Effusion	8 (21.6)	1 (2.7)	9 (24.3)
Synovial hypertrophy	5 (13.5)	0	5 (13.5)
Doppler	3 (8.1)	0	3 (8.1)
**Inferior recess**			
Effusion	7 (18.9)	3 (8.1)	10 (27)
Synovial hypertrophy	3 (8.1)	1 (2.7)	4 (10.8)
Doppler	1 (2.7)	0	1 (8.1)
**Posterior recess**			
Effusion	12 (32.4)	3 (8.1)	15 (40.5)
Synovial hypertrophy	11 (29.7)	3 (8.1)	14 (37.8)
Doppler	4 (10.8)	1 (2.7)	5 (13.5)
**SAD bursea**			
Bursitis	8 (21.6)	6 (16.2)	14 (37.8)
Doppler	4 (10.8)	2 (5.4)	6 (16.2)

**Table 3:** Association between ultrasonography abnormalities and clinical and biological features

	No US Abnormality	US Abnormality	p	No Effusion	Effusion	p	No Synovitis	Synovitis	p	No Doppler	Dpppler	p	No Bursitis	Bursitis	p
Age (years)	51.68±10.17	43.17±13.2	0.08	48.24±10.87	52.05±11.03	0.29	46.48±8.34	55.31±12.21	**0.01**	48.76±9.6	63±14.7	**0.01**	50.52±12.23	49.93±8.94	0.87
Gender (Female)	26 (81.3)	6 (18.8)	0.56	15 (46.9)	17 (53.1)	1.00	19 (59.4)	13 (40.6)	0.63	29 (90.6)	3 (9.4)	0.45	19 (59.4)	13 (40.6)	0.63
Disease duration (months)	9 [5;22]	1 [0.6;5.5]	**0.01**	11 [2;12]	9 [4;22]	0.30	7 [3;22]	8 [3;13]	0.82	8.5[4.3;21.5]	1.5 [1;4.3]	**0.02**	7 [3;18]	9 [3;27]	0.47
VAS pain	51.61±21.77	50±17.88	0.86	53.52±23.16	49.5±19.32	0.56	54.76±24.41	46.87±14.93	0.23	52.42±21.51	42.50±15	0.37	49.56±19.18	54.28±24.08	0.51
DAS28	5.05±1.52	5.11±1.51	0.94	4.98±1.39	5.13±1.61	0.78	5.29±1.42	4.77±1.56	0.32	5±1.5	5.5±1.7	0.56	5.41±1.46	4.44±1.38	0.07
SDAI	21 [17;43]	34 [22;61]	0.19	28 [18;55]	36 [22;69]	0.42	31 [23;54]	30 [18;66]	0.67	29.5 [19.6;53.6]	93[18; 155]	**0.006**	44 [24;74]	22 [18; 30]	**0.02**
CDAI	20.61±14	20.83±9.23	0.97	18.94±11.21	22.1±14.87	0.47	22.61±13.78	18.06±12.42	0.31	20.48±12.86	22±18.31	0.83	22.47±1331	17.64±13.03	0.28
HAQ	1.2±0.7	1.2±0.5	0.99	1.08±0.58	1.22±0.75	0.53	1.10±0.61	1.24±0.77	0.55	1.16±0.69	1.15±0.62	0.98	1.3±0.6	0.93±0.74	0.12
RF (U/l)	76 [22;136]	68 [28;170]	0.45	60 [27;124]	119[60;237]	0.08	68 [28;160]	85 [27;256]	0.75	68 [28;160]	286 [60;]	0.38	66 [28;136]	70 [26;256]	0.74
ACPA (U/l)	28 [11;2392]	200 [125;334]	0.32	200 [200;348]	200[92;334]	0.91	200[60;373]	182[56;325]	0.45	200 [40;350]	200 [163;]	0.97	200[39;356]	205[131;290]	0.67
ESR (mm)	36 [17;82]	39 [21;68]	0.74	37[26;65]	40[17;68]	0.80	40 [31;66]	37 [14;72]	0.45	36 [18;64]	59 [42;77]	0.13	40[31;79]	32[14; 44]	**0.05**
CRP (mg/l)	5 [2;14]	11 [4;33]	0.18	10 [5;24]	12 [2;32]	0.98	10 [28;160]	9 [3;37]	1.00	10 [4;23]	56 [9;126]	0.14	21[5;36]	7[2; 11]	**0.03**
Shoulder pain	31 (83.8)	6 (16.2)	0.67	9 (53.3)	13 (59.1)	0.45	14 (63.6)	8 (36.4)	0.30	20 (90.9)	2 (9.1)	1.00	13 (59.1)	9 (40.9)	0.64
Shoulder limitation	15 (83.3)	3 (16.7)	1.00	9 (50)	9 (50)	0.63	12 (66.7)	6 (33.3)	0.23	15 (83.3)	3 (16. 7)	0.34	14 (77.8)	4 (22.2)	**0.05**

## Discussion

In this study which concerned 37 RA patients, US abnormalities were found in 83.8% of patients; bilateral in 59.5% of them. The most frequent US finding was erosion (64.9%) followed by effusion (54.1%) and synovial hypertrophy (43.2%) in GHJ, SAD bursitis (37.8%) and Doppler signal in GHJ (10.8%). The posterior recess was the most affected (54.1%) with: Effusion in 40.5% of patients, SH in 37.8% and Doppler in 13.5% of cases. Prevalences in the current study were higher than reported in a sample of healthy subjects in which GHJ was less frequently involved. In particular, SE was found only in 2.6% and detected only at the level of the posterior recess only in patients aged up to 50 years. In this sample of healthy subjects, SE within SAD bursa was present in 11.3% with SH only in 1 shoulder without Doppler signal [10]. The frequency of abnormal US findings of rheumatoid shoulder joints differs depending on the patient population studied. In a study evaluating 44 hospitalized RA patients with mean disease duration of 12 years, subacromial bursitis was the most frequent finding, followed by GH joint synovitis [11]. In another US study evaluating 100 patients with RA with a mean disease duration of 4.5 years, 14 cases presented with involvement of the GHJ and 22 with inflammatory abnormalities of the SAD bursa [12]. Yet, our results of prevalence remain higher possibly related to the short disease duration in our sample although the majority of patients were on treatment. In a study comparing patients with RA and patients with degenerative shoulder disease, combination of glenohumeral joint effusion, bone cartilage reduction and humeral erosions was able to identify patients with RA in a population of patients with painful shoulder disease with a moderately high degree of confidence [13]. Moreover, Strunk et al showed that power Doppler sonography helps to differentiate between degenerative shoulder disorders and rheumatoid shoulder [14]. When we assessed the sites of inflammation in painful rheumatoid shoulder by MS ultrasound and power Doppler, the most common US finding was effusion or synovitis in 59% of painful GHJ accompanied or not with subdeltoid bursitis, detectable in the posterior scan in 87% of shoulders. US joint erosions on the humeral head were detected in 59% of joints with longer disease duration [15]. This is partially consistent with our results. Assessing different US inflammatory findings in relation to clinical and biological characteristics of patients plays an important role in providing information for practitioners regarding which features or variables are associated with US abnormalities in patients suffering or not from shoulder pain. In our study, patients with US abnormalities at the shoulders had shorter disease duration. In particular, SH was associated with advanced age, and signal Doppler with advanced age, shorter disease duration and with higher disease activity assessed by SDAI. SDAI as an index of RA activity was been shown superior over DAS28 by Alejandro Balsa et al [16].

Abnormalities in anterior recess were associated with elevated synovial index and rheumatoid factor level. Presence of SAD bursitis was not linked to disease activity. In a study of G. Sakellariou et al, patients with US inflammatory involvement had a longer median disease duration, were more frequently RF pos¬itive, had a higher disease activity and higher acute phase reactants, a higher level of disability and more pain, with an increased frequency of spontaneous shoulder pain and higher median VAS pain [12]. Our findings underline the findings of the literature concerning the prevalence of US abnormalities at the shoulder and their association with clinical disease features, especially with disease activity. These findings are in keeping with previous works that identified a relation between shoulder involvement and disease activity and disability [17, 18], even though some limitations. The cross-sectional design does not allow to evaluate the impact of US findings on relevant outcomes, and the univariate analysis does not take into account the potential presence of confounders. Also, the results are based on a small population owing to the short duration of patients’ enrolling. However, this study objectifies the role of US in the assessment of large joint such shoulder in RA patients.

## Conclusion

Ultrasound allows the differentiation between degenerative shoulder lesions and signs of disease activity in RA and identifies a subgroup of patients with higher disease activity that could benefit from a more aggressive treatment approach. Further studies are needed to investigate these findings.

### What is known about this topic

- All joints can be affected during the natural history of Rheumatoid Arthritis (RA);
- Clinical evaluation and conventional radiography remain insufficient for assessing several structures;
- Musculoskeletal ultrasonography has a great role in detecting subclinical abnormalities in rheumatoid joints in order to achieve a low disease status and eventually remission.

### What this study adds

- This study tries to assess the prevalence of abnormalities in rheumatoid shoulder by ultrasonography and to identify risk factors associated with.
